# Elevated Serum Human Epididymis Protein 4 Is Associated With Disease Activity and Systemic Involvements in Primary Sjögren’s Syndrome

**DOI:** 10.3389/fimmu.2021.670642

**Published:** 2021-06-23

**Authors:** Jiali Chen, Feng Sun, Huizhang Bao, Liu Liang, Minghua Zhan, Haihong Yao, Jing He, Yudong Liu

**Affiliations:** ^1^ Department of Rheumatology and Immunology, Peking University People’s Hospital, Beijing, China; ^2^ Department of Clinical Laboratory, Peking University People’s Hospital, Beijing, China

**Keywords:** Sjögren’s syndrome, human epididymis protein 4 (HE4), tumor biomarker, interstitial lung disease, clinical stratification

## Abstract

**Background:**

We aimed to investigate the clinical utility of human epididymis protein 4, a tumor biomarker being widely utilized in clinical practice in the diagnosis of ovarian cancer, in primary Sjögren’s Syndrome (pSS).

**Methods:**

A total of 109 pSS patients and 113 healthy controls (HCs) were included in the study. HE4 were determined by Roche Cobas E601 electrochemical luminescence analyzer. Clinical and laboratory findings were reviewed, and the relationships between HE4 and clinical parameters were determined by Spearman’s correlation test. The European league against rheumatism Sjögren’s syndrome disease activity index (ESSDAI) was utilized to evaluate disease activity.

**Findings:**

The levels of HE4 were significantly elevated in patients with pSS compared to HCs (103.65 pmol/L *vs.* 46.52 pmol/L, *p*<0.001). The levels of HE4 were positively correlated with ESSDAI scores (*r*=0.462, *p*<0.001). Significant positive correlations between the levels of HE4 with pulmonary involvements (*r*=0.442, *p*<0.001) and renal involvements (*r*=0.320, *p*=0.001) were observed. Receiver operating curve (ROC) analysis revealed an optimal cut-off value of 104.90 pmol/L and 128.05 pmol/L for distinguishing patients with pulmonary and renal involvements, with the areas under the ROC curve (AUCs) of 0.778 (95%CI 0.685-0.870, *p*<0.001) and 0.768 (95%CI 0.646-0.891, *p*=0.001), respectively. Among patients with pulmonary involvement, the levels of HE4 were positively correlated with the semiquantitative HRCT grade (*r*=0.417, *p*=0.016), and negatively correlated with the percentage of forced vital capacity (FVC) (*r*= -0.460, *p*=0.047) and diffusing capacity of the lung for carbon monoxide (DLco) (*r*= -0.623, *p*=0.004). For patients with renal involvement, HE4 was positively correlated with creatinine (*r*=0.588, *p*=0.021) and negatively correlated with estimated glomerular filtration rate (*r*= -0.599, *p*=0.030).

**Conclusions:**

Our findings demonstrated a novel role of HE4 in clinical stratification of pSS, suggesting that introducing HE4 to the current pSS test panel may provide additional diagnostic value, particularly in evaluating disease activity and pulmonary/renal involvements.

## Introduction

Primary Sjögren’s Syndrome (pSS) is a chronic multifactorial autoimmune disorder with a female-to-male predominance of 9 to 1 (1, 2). The hallmark of the disease is lymphocytic infiltration in the exocrine glands, leading to progressive loss of the secretory function. In addition to glandular features, approximately 30-40% of patients with pSS develop a wide spectrum of extra-glandular complications, including hematological, pulmonary or renal manifestations (2), rendering this subgroup of pSS patients at risk for higher mortality and morbidity (3). Biomarkers have been considered as a useful tool to stratify the complex and heterogeneous phenotypes in pSS. Whereas there is growing interest in developing biomarkers reflecting disease activity and risk stratification in pSS (4), few biomarkers have been utilized in clinical practice.

Human epididymis protein 4 (HE4), a human epididymis-specific protein, has been widely utilized as a tumor biomarker in the diagnosis of ovarian cancer in clinical practice (5). Serum HE4 testing was recently introduced into our tumor biomarker screening panel for females in routine clinical practice. Patients with chronic inflammation and autoimmune responses are at increased risk of malignancy (6). As such, most inpatients with rheumatoid diseases are routinely screened for malignancy utilizing a tumor biomarker screening panel. During routine clinical practice with this panel, we observed that the levels of HE4 were elevated in patients with pSS. Of interest, those pSS patients with high levels of HE4 did not display any signs of malignancy in the follow-up computerized tomography (CT) scan.

Of interest, HE4 is expressed in multiple tissues in the oral cavity, the respiratory tracts as well as in renal tubular epithelial cells (7) and has been shown elevated in patients with renal fibrosis (8) and cystic fibrosis (9). The increased levels of HE4 in pSS patients propel us to investigate the clinical utility of this biomarker in pSS. In this study, we found that active pSS patients displayed significantly higher levels of HE4 compared to inactive pSS patients, and the levels of HE4 were significantly correlated with the disease activity scores. Further, we identified a significant positive correlation between HE4 and pulmonary involvement and renal involvement. Our findings shed light on a novel role of HE4 in disease stratification in pSS.

## Materials and Methods

### Subjects

On Jan. 2020, HE4 was added to the female-tumor biomarker screening panel, which consists of 10 tumor biomarkers, including alpha fetoprotein (AFP), carcinoembryonic antigen (CEA), neuron-specific enolase (NSE), cytokeratin 19 fragment antigen 21-1 (CYFRA21-1), cancer antigen 125 (CA125), CA15-3, CA19-9, progastrin-releasing peptide (ProGRP), squamous cell carcinoma (SCC), and HE4. All consecutive female pSS inpatients who were tested for the female-tumor biomarker screening panel from Jan. 2020 to Mar. 2021 were reviewed. Serum from 17 male pSS inpatients were also collected and tested for the panel. pSS was diagnosed according to the 2002 American-European Consensus Group (AECG) classification criteria (10) and patients with cancer or a history of malignant neoplasm were excluded. Gender and age-matched healthy controls (HCs) with no evidence of autoimmune disease, malignancy or other diseases were included. Female HCs were individuals who went to Peking University People’s Hospital (PKUPH) for annual physical examination and were tested for the female-tumor biomarker screening panel. Male HCs were individuals who went to PKUPH for annual physical examination. Serum from male HCs were collected and tested for the panel. The study protocol was reviewed and approved by the Ethical Committee of PKUPH (Protocol number: 2019PHB244) and all participants gave their written consent to the study.

### Data Collection

Demographic features and clinical characteristics were collected, including gender, age, disease duration, clinical symptoms and systemic manifestations. Systemic features were described and assessed according to the European League Against Rheumatism (EULAR) Sjögren’s syndrome disease activity index (ESSDAI), which includes 12 domains. Active disease was define as ESSDAI ≥5 points (11). Laboratory findings included blood cell counts, including leukocyte, lymphocyte, hemoglobin and platelet, biochemistry parameters (serum potassium, creatinine, glomerular filter rate), erythrocyte sedimentation rate, gamma-globulin, immunoglobulins G, complement (C3, C4), cryoglobulin, antibodies (rheumatoid factor, anti-nuclear antibody, anti-SSA antibody, anti-SSB antibody, anti-mitochondrial antibody, anti-thyroglobulin antibody, anti-thyroid peroxidase antibody).

### Assessment of Pulmonary Involvement

The SS-related pulmonary disease mainly presented as interstitial lung disease (ILD), which included the specific types of lymphocytic interstitial pneumonia (LIP), non-specific interstitial pneumonia (NSIP), usual interstitial pneumonitis (UIP), bronchiolitis obliterans and organizing pneumonia (12). The assessment of ILD was performed by two pulmonologists and two radiologists with more than 10 years of thoracic imaging experience, which was based on the symptoms, mainly included persistent cough, breathlessness or dyspnea due to bronchial involvement, and respective abnormalities suggestive of ILD in high-resolution computed tomography (HRCT) and pulmonary function tests (PFTs).

The SS-related ILD was evaluated semi-quantitatively based on the HRCT scans, and any indeterminate ILD were excluded from the analyses. A Likert scale (grade 1, 1–25%; grade 2, 26–50%; grade 3, 51– 75%; grade 4, 76–100%) was used to assess the extent of parenchymal abnormality, including pure ground-glass opacity, lung fibrosis, honeycombing, and emphysema (13). Each of three lung zones as indicated below was scored: upper, extending from apex to aortic arch; middle, from aortic arch to inferior pulmonary veins; and lower, from inferior pulmonary veins to diaphragm (13).

PFTs were performed among patients with ILD and the following indexes were recorded including forced vital capacity (FVC), the median forced expiratory volume in 1 second (FEV1), and diffusing capacity of the lung for carbon monoxide (DLco). FVC, FEV1 and DLco were described as a percentage of the predicted values for the patient’s age, sex, and height (14). Abnormalities of PFTs were defined as predicted values of forced vital capacity (FVC) < 80% and diffusing capacity of the lung for carbon monoxide (DLco) < 70% (13).

### Assessment of Renal Involvement

Renal involvement was assessed as previously described by Brito-Zeron (12). Briefly, tubulointerstitial nephritis caused by type I renal tubular acidosis (RTA) is the major manifestation of renal involvement in patients with SS (12). According to the European League Against Rheumatism (EULAR) recommendation, the diagnosis of RTA was mainly based on the symptoms of hypokalemic weakness, and persistent urine pH > 5.3 even in the presence of metabolic acidosis induced by NH4^+^Cl loading (15). In contrast to tubular involvement, SS-related glomerulonephritis was rarely reported and patients always combined with positive cryoglobulin. In addition, patients could have proteinuria (between 0.5 to 1g/day) and without hematuria or renal failure (16).

### Serum Tumor Biomarkers Determination

All the ten tumor biomarkers in the female-tumor biomarker screening panel were determined by Roche Cobas E601 electrochemical luminescence analyzer (Hoffmann-La Roche AG., Basel, Switzerland) in the Department of Clinical Laboratory of PKUPH, which is accredited by College of American Pathologists (CAP).

### Statistical Analysis

Continuous variables were presented as mean ± standard deviation (SD) for normal distribution or median (interquartile range, IQR) for abnormal distribution. Categorical variables were shown as numbers (percentages) of the total samples. The statistical significance between groups was assessed using the Mann-Whitney U test, Student t-test, Chi-square (χ^2^) test, where it was applicable. The receiver operating characteristic (ROC) curve was generated to evaluate the sensitivity, specificity, Youden index and areas under the ROC curve (AUC) with the 95% confidence interval (CI). Spearman’s correlation test was used to determine the relationships between HE4 and clinical parameters. Data analyses were calculated using SPSS 24.0 statistical software package (SPSS Inc., Chicago, Illinois, USA) or GraphPad Prism 8 (GraphPad Software Inc.). A significant difference was defined as *p*<0.05.

## Results

### Characteristics of Patients With pSS

A total of 109 pSS patients and 113 gender- and age-matched HCs were analyzed in our study. The median age and disease duration was 62 [Interquartile Range (IQR) 51, 68] and 6 (3, 11) years, respectively. The median disease activity index of ESSDAI was 5 (IQR 3, 10). A total of 60 (55.0%) patients had active disease, which defined as ESSDAI≥5. Among pSS patients, the most common systemic manifestations were hematological (49, 45.0%), pulmonary (33, 30.3%), articular (25, 22.9%), and renal involvement (15, 13.8%). For pulmonary involvement, patients mainly presented as ILD, including LIP (16, 14.7%) and NSIP (17, 15.6%). For renal involvement, there were 12 (11.0%) patients with tubulointerstitial nephritis and 3 (2.8%) patients with glomerulonephritis. Detailed clinical characteristics were summarized in **Table 1**.

**Table 1:** Demographic and clinical characteristics between patients with primary Sjögren’s syndrome (pSS) and healthy controls (HCs).

Variables	pSS (n=109)	HCs (n=113)	*p*-value
Gender, Female/Male	92 (84.4)/17 (15.6)	95 (84.1)/18 (15.9)	0.946
Age, years	62 (51, 68)	61 (52, 69)	0.304
Duration, years	6 (3, 11)		
Xerostomia	89 (81.7)		
Xerophthalmia	92 (84.4)		
Dental caries	32 (29.4)		
Constitutional symptom	12 (11.0)		
Lymphadenopathy	13 (11.9)		
Glandular swelling	22 (20.2)		
Systemic involvements			
Articular	25 (22.9)		
Cutaneous	6 (5.5)		
Pulmonary	33 (30.3)		
LIP	16 (14.7)		
NSIP	17 (15.6)		
Renal	15 (13.8)		
Tubulointerstitial nephritis	12 (11.0)		
Glomerulonephritis	3 (2.8)		
Muscular	4 (3.7)		
Peripheral neuropathy	6 (5.5)		
Central neuropathy	1 (0.9)		
Hematological	49 (45.0)		
Leukocyte, 10^9/L	4.8 (3.5, 7.6)		
Lymphocyte, 10^9/L	1.3 (1.1, 1.9)		
Hemoglobin, g/L	125 (115, 140)		
Platelet, 10^9/L	198 (136, 241)		
Potassium, mmol/L	3.75 (3.56, 4.02)		
Urea, mmol/L	5.47 (4.50, 6.64)		
Creatinine, umol/L	64.0 (57.0, 79.8)		
eGFR, ml/min*1.73m^2^	87.6 (66.0, 98.8)		
ESR, mm/h	21 (8, 44)		
Gama-globulin, %	20.6 (18.0, 27.2)		
Immunoglobulin G, g/L	15.5 (12.6, 20.1)		
Low complement 3	27 (24.8)		
Low complement 4	26 (23.9)		
Rheumatoid factor, positive	33/64 (51.6)		
ANA, positive	72/101 (71.3)		
Anti-SSA, positive	69/106 (65.1)		
Anti-SSB, positive	43/106 (40.6)		
Focal lymphocytic sialadenitis, positive	5/7 (71.4)		
Anti-mitochondrial antibody, positive	15/97 (15.5)		
Cryoglobulin, positive	6/18 (33.3)		
Anti-thyroglobulin antibody, positive	39/74 (52.7)		
Anti-thyroid peroxidase antibody, positive	24/74 (32.4)		
Medications			
HCQ	29 (26.6)		
Corticosteroids	44 (40.4)		
Immunosuppressive agents	34 (31.2)		
ESSDAI	5 (3,10)		
ESSDAI≥5	60 (55.0)		

Data are presented as median (Interquartile Range) and n (%).

ANA, anti-nuclear antibody; ESR, Erythrocyte Sedimentation Rate; ESSDAI, European league against rheumatism (EULAR) Sjögren’s syndrome disease activity index; HCQ, Hydroxychloroquine; LIP, lymphocytic interstitial pneumonia; NSIP, nonspecific interstitial pneumonia.

### The Levels of HE4 Were Significantly Elevated in Patients With pSS

The levels of all biomarkers in the female-tumor biomarker screening panel between pSS patients and HCs are shown in **Table 2**. Compared to HCs, the levels of CEA, CA15-3, CYFRA21-1, ProGRP, SCC, and HE4 were significantly elevated in patients with pSS. In particular, the median level of HE4 in pSS patients was approximately 2.5 times higher than that of HCs (103.65 pmol/L *vs* 46.52 pmol/L, p<0.001).

**Table 2:** Levels of tumor markers between patients with primary Sjögren’s Syndrome (pSS) and healthy controls (HCs).

Marker	pSS (n=109)	HCs (n=113)	*p*-value
CEA, ng/mL	1.62 (1.06, 3.64)	1.38 (0.95, 1.84)	0.011
AFP, ng/mL	2.64 (2.26, 3.06)	2.58 (2.01, 3.72)	0.557
CA19-9, U/mL	14.28 (7.34, 34.01)	10.11 (7.28, 16.80)	0.165
CA125, U/mL	16.92 (12.43, 30.09)	12.83 (8.94, 18.51)	0.105
CA15-3, U/mL	12.18 (9.14, 18.65)	7.39 (6.27, 10.08)	<0.001
CYFRA21-1, ng/mL	2.65 (2.11, 3.52)	1.68 (1.30, 2.37)	<0.001
NSE, ng/mL	11.43 (9.63, 13.71)	11.72 (9.94, 13.74)	0.060
ProGRP, pg/mL	30.40 (28.25, 39.95)	36.50 (29.85, 44.10)	0.012
SCC, ng/mL	1.03 (0.79, 1.42)	1.07 (0.81, 1.35)	<0.001
HE4, pmol/L	103.65 (64.35, 178.70)	46.52 (40.05, 52.66)	<0.001

Data are presented as median (Interquartile Range) and n (%). Statistical significance was determined by Mann-Whitney U test and Chi-square (χ^2^) test.

AFP, alpha fetoprotein; CA125, cancer antigen 125; CA15-3, cancer antigen 15-3; CA19-9, cancer antigen 19-9; CEA, carcino-embryonic antigen; CYFRA21-1, cytokeratin 19 fragment antigen 21-1; HE4, Human epididymis protein 4; IQR, Interquartile Range; NSE, neuron-specific enolase; ProGRP, progastrin-releasing peptide.

The threshold for positivity of HE4 was set at 75.23 pmol/L based on the upper limit of 95% confidence interval of HCs. Patients in the HE4-positive group showed significantly lower levels of hemoglobin (*p*<0.001) and higher levels of ESR compared to HE4-negative patients (*p*<0.001). In addition, patients in the HE4-positive group displayed significantly higher disease activity index than the HE4-negative group [8 (4, 14) *vs* 4 (3, 7), *p*<0.001]. Specifically, the proportion of patients with ESSDAI≥5 in the positive group was twice as much as that in the negative group (73.7% *vs* 34.6%, *p*<0.001). Further, patients in the HE4-positive group had higher proportion of xerostomia (89.5% *vs* 73.1%, *p*=0.027) and xerophthalmia (93.0% *vs* 75.0%, *p*=0.047). Of note, patients in the HE4-positive group exhibited a significantly higher percentage of systemic involvement than the negative group, including pulmonary (45.6% *vs* 13.5%, *p*<0.001) and renal involvements (22.8% *vs* 3.8%, *p*=0.004) (**Table 3**).

**Table 3:** Clinical characteristics of patients with primary Sjögren’s Syndrome (pSS) between HE4-nagetive and positive groups*.

Variables	HE4-negative (n=52)	HE4-positive (n=57)	*p-*value
Gender, Female	42 (80.8)	50 (87.7)	0.318
Age, years	56 (38, 64)	64 (60, 70)	<0.001
Duration, years	6 (3, 10)	7 (3, 12)	0.135
Xerostomia	38 (73.1)	51 (89.5)	0.027
Xerophthalmia	39 (75.0)	53 (93.0)	0.047
Constitutional symptom	6 (11.5)	6 (5.0)	0.892
Lymphadenopathy	5 (9.6)	8 (15.0)	0.477
Glandular swelling	13 (25.0)	9 (17.5)	0.231
Systemic involvements			
Articular	11 (21.2)	24 (27.5)	0.673
Cutaneous	3 (5.8)	3 (10.0)	1
Pulmonary	7 (13.5)	26 (45.6)	<0.001
LIP	5 (9.6)	11 (19.3)	0.225
NSIP	2 (3.8)	15 (26.3)	0.225
Renal	2 (3.8)	13 (22.8)	0.004
Tubulointerstitial nephritis	2 (3.8)	10 (17.5)	0.022
Glomerulonephritis	1 (1.9)	2 (3.5)	1.000
Muscular	1 (1.9)	3 (5.3)	0.62
Peripheral neuropathy	3 (5.8)	3 (5.3)	1.00
Central neuropathy	0 (0.0)	1 (1.8)	0.168
Hematological	19 (36.5)	30 (52.6)	0.092
Leukocyte, 10^9/L	4.9 (4.0, 8.0)	4.2 (3.2,7.2)	0.903
Lymphocyte, 10^9/L	1.3 (1.1, 2.1)	1.3 (1.0, 1.9)	0.527
Hemoglobin, g/L	129 (123, 142)	116 (108,126)	<0.001
Platelet, 10^9/L	193 (146, 238)	179 (120,240)	0.616
ESR, mm/h	10 (7, 26)	25 (16,53)	<0.001
Gama-globulin, %	20.0 (17.6, 24.7)	21.1 (18.1, 33.8)	0.119
Immunoglobulin G, g/L	15.7 (11.8, 20.1)	15.5 (12.9, 24.0)	0.569
ESSDAI	4 (3, 7)	8 (4, 14)	<0.001
ESSDAI≥5	18 (34.6)	42 (73.7)	<0.001

*The reference range of human epididymis protein 4 (HE4) was determined by the upper limit of 95% confidence interval of healthy controls.

Data are presented as median (Interquartile Range) and n (%). Statistical significance was determined by Mann-Whitney U test and Chi-square (χ^2^) test. ESR, Erythrocyte Sedimentation Rate; ESSDAI, European league against rheumatism (EULAR) Sjögren’s syndrome disease activity index; HCQ, Hydroxychloroquine; LIP, lymphocytic interstitial pneumonia; NSIP, nonspecific interstitial pneumonia.

### Associations Between the Levels of HE4 and ESSDAI

**Figure 1:**
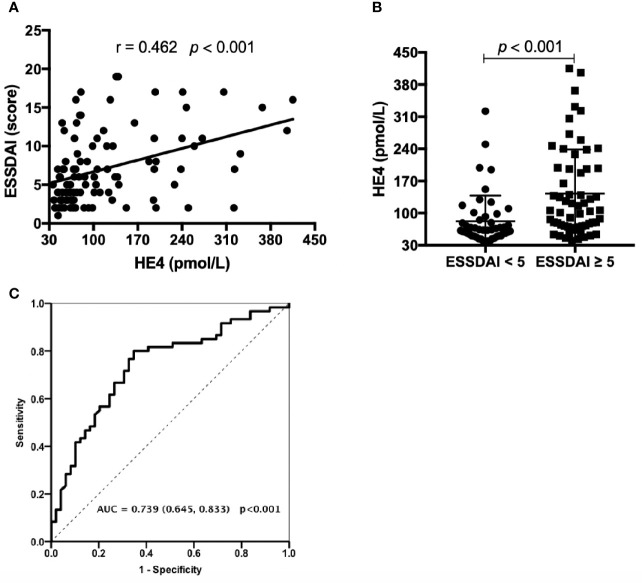
Associations between the levels of HE4 and ESSDAI. **(A)** Associations between the levels of HE4 and ESSDAI. **(B)** Levels of HE4 were significantly higher in patients with active disease (ESSDAI≥5, n = 60) than inactive disease (ESSDAI<5, n = 49). **(C)** Receiver operating curve analysis (ROC) on the clinical performance of HE4 in identifying patients with active disease. Statistical significance was determined by Mann-Whitney U test, Spearman’s correlation test and ROC analysis. ESSDAI, European league against rheumatism Sjögren’s syndrome disease activity index.

Significant correlations between the levels of HE4 and ESSDAI scores were observed (*r*=0.462, *p*<0.001) (**Figure 1A**). Patients with active disease displayed significantly higher levels of HE4 compared to inactive patients (*p*<0.001) (**Figure 1B**). ROC analysis was utilized to characterize the clinical performance of HE4 in identifying patients with active disease. Based on the Youden index of 0.453, the optimal cut-off value of HE4 in distinguishing active disease from inactive disease was 69.50 pmol/L with a sensitivity of 80.0% and specificity of 65.3% with an AUC of 0.739 (95%CI 0.645-0.833, *p*<0.001) (**Figure 1C**).

Serum level of HE4 was positively correlated with age (*r*=0.598, *p*<0.001), disease duration (*r*=0.297, *p*=0.006), xerostomia (*r*=0.345, *p*<0.001) and xerophthalmia (*r*=0.257, *p*=0.007), ESR (*r*=0.565, *p*<0.001) and negatively correlated with hemoglobin (*r*=-0.444, *p*<0.001). No significant correlations between HE4 and γ-globulin, IgG, C3, C4 and rheumatoid factor were identified (**Table 4**).

**Table 4:** Correlations between HE4 and characteristics of patients with pSS.

Clinical parameters	*r*	*p-*value	Domains of ESSDAI	*r*	*p-*value
Age	0.598	<0.001	Constitutional	0.009	0.923
Duration	0.297	0.006	Lymphadenopathy	0.096	0.322
Xerostomia	0.345	<0.001	Glandular swelling	-0.079	0.413
Xerophthalmia	0.257	0.007	Articular	-0.025	0.797
Leukocyte	0.103	0.298	Cutaneous	-0.043	0.654
Lymphocyte	-0.006	0.948	Pulmonary	0.442	<0.001
Hemoglobin	-0.444	<0.001	Renal	0.320	0.001
Platelet	-0.091	0.354	Muscular	0.029	0.767
ESR	0.565	<0.001	Peripheral nervous system	-0.047	0.628
Gama-globulin	0.141	0.197	Central nervous system	0.011	0.912
Immunoglobulin G	0.021	0.835	Hematological	0.112	0.246
Complement 3	0.079	0.426	Biological	0.020	0.839
Complement 4	0.154	0.120			
Rheumatoid factor	0.047	0.710			

Statistical significance was determined by Spearman’s correlation test.

ESR, Erythrocyte Sedimentation Rate; ESSDAI, European league against rheumatism (EULAR) Sjögren’s syndrome disease activity index; HE4, Human epididymis protein 4.

### Associations Between the Levels of HE4 and Systemic Involvements

The relationships between the levels of HE4 and each ESSDAI domain were investigated. Significant positive correlations between the levels of HE4 with pulmonary involvements (*r*=0.442, *p*<0.001) and renal involvements (*r*=0.320, *p*=0.001) were observed. However, there were no significant correlations between the levels of HE4 and other domains (**Table 4**).

**Figure 2:**
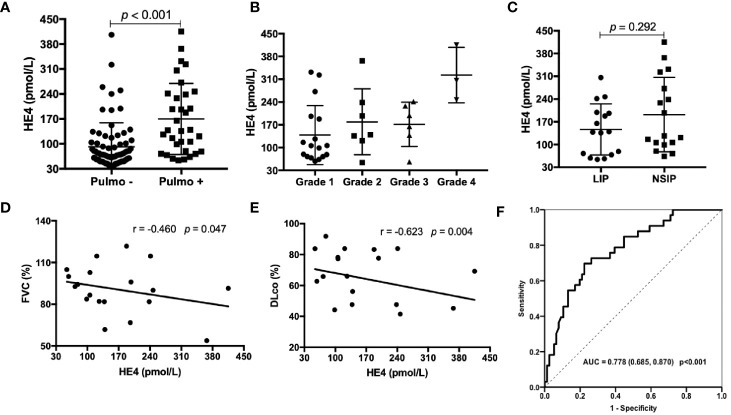
Associations between the levels of HE4 and pulmonary involvements. **(A)** Patients with pulmonary involvement (n = 33) had higher levels of HE4 than those without pulmonary involvement (n = 76). **(B)** Serum levels of HE4 in patients with ILD according to semiquantitative CT grades: grade 1 (n = 17); grade 2 (n = 7); grade 3 (n = 6); grade 4 (n =3 ). **(C)** Serum HE4 was similar between the groups of LIP (n = 16) and NSIP (n=17). **(D, E)** HE4 was negatively correlated with FVC% and DLCO% (n = 19). **(F)** Receiver operating curve (ROC) analysis on the clinical performance of HE4 in identifying patients with pulmonary involvement. Statistical significance was determined by Mann-Whitney U test, Spearman’s correlation test and ROC analysis. Pulmo, pulmonary; LIP, lymphocytic interstitial pneumonia; NSIP, nonspecific interstitial pneumonia; FVC, forced vital capacity; DLco, diffusing capacity of the lung for carbon monoxide.

pSS patients with pulmonary involvements showed significantly higher levels of HE4 compared to those without pulmonary involvements (**Figure 2A**, *p*<0.001). pSS-ILD patients were divided into 4 groups based on the semiquantitative HRCT grade (Grade 1 to Grade 4). The CT grade positively correlated with serum level of HE4 (*r*=0.417, *p*=0.016) and patients with grade 4 had higher levels of HE4 than other grades (**Figure 2B**). No significant differences between LIP and NSIP were observed (*p*=0.292, **Figure 2C**). Significantly negative correlations between the levels of HE4 and percentage of FVC (*r*= -0.460, *p*=0.047) and DLco (*r*= -0.623, *p*=0.004) were noticed (**Figures 2D, E**) . ROC analysis revealed a cut-off value of 104.90 pmol/L (Youden Index of 0.473) with a sensitivity of 69.7%, a specificity of 77.6% and an AUC of 0.778 (95%CI 0.685-0.870, *p*<0.001) in distinguishing pSS patients with pulmonary involvements from pSS patients without pulmonary involvements (**Figure 2F**).

**Figure 3:**
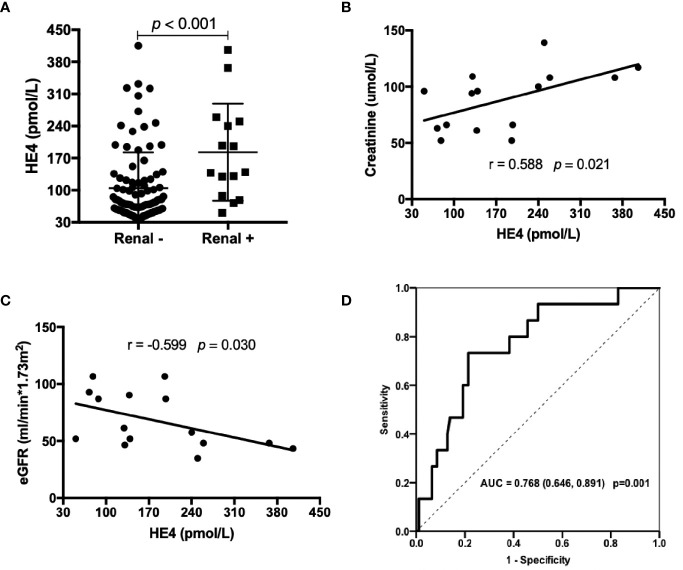
Associations between the levels of HE4 and renal involvements. **(A)** Patients with renal involvement (n=15) had higher levels of HE4 than those without renal involvement (n = 94). **(B, C)** Serum levels of HE4 was positively correlated with creatinine and negatively correlated with eGFR (n = 15). **(D)** Receiver operating curve (ROC) analysis on the clinical performance of HE4 in identifying patients with renal involvement. Statistical significance was determined by Mann-Whitney U test, Spearman’s correlation test and ROC analysis. eGFR, estimated glomerular filtration rate.

pSS patients with renal involvements displayed significantly higher levels of HE4 compared to those without renal involvements (**Figure 3A**, *p*<0.001). HE4 was positively correlated with serum creatinine (*r*=0.588, *p*=0.021, **Figure 3B**) and negatively correlated with estimated glomerular filtration rate (*r*= -0.599, *p*=0.030, **Figure 3C**). ROC analysis revealed a cut-off value of 128.05 pmol/L (a Youden Index of 0.52) with a sensitivity of 73.3%, a specificity of 78.7% and an AUC of 0.768 (95%CI 0.646-0.891, *p*=0.001) in distinguishing pSS patients with renal involvements from pSS patients without renal involvements (**Figure 3D**).

## Discussion

In this retrospective study, by utilizing the real-world data from clinical practice, we identified a novel role for serum HE4 in clinical stratification in patients with pSS. HE4, a tumor biomarker approved by the US Food and Drug Administration (FDA) in 2009 and by the National Medical Products Administration (NMPA) (formerly the China FDA) in 2012 for the routine diagnostics of ovarian cancer, was found significantly elevated in patients with pSS and correlated with disease activity. Further, we found significant positive correlations between the levels of HE4 with pulmonary involvements and renal involvements. As far as we know, few studies, if any, have investigated the role of HE4 in the diagnosis of pSS. Given the clinical need of biomarkers for assessing disease activity and identifying extra-glandular involvements, our findings thus represent an important endeavor in clinical subtyping of patients with pSS.

HE4 was first identified as a secreted, epididymis-specific protein (17), and later was found to be expressed in multiple tissues in the oral cavity, the respiratory tracts as well as in renal tubular epithelial cells (7). Although previous work on HE4 has primarily focused on its clinical utility as a tumor biomarker, increasing evidence has suggested that HE4 may have diagnostic potential in other clinical settings, including lung adenocarcinomas (18), renal fibrosis (8) and cystic fibrosis (9). Of interest, the levels of HE4 were upregulated in human and mouse fibrotic kidneys and were elevated in the serum of patients with kidney fibrosis (8). In addition, elevated levels of HE4 mRNA, as well as HE4 protein were detected in lung biopsies of patients with cystic fibrosis (9, 18). Very recently, Zhang et al. reported that serum HE4 levels were significantly increased in patients with systemic sclerosis (SSc)-ILD compared to SSc-non-ILD, which was consistent with our findings (19). Nishiyama et al. also showed that HE4 was a new biomarker to predict the prognosis of progressive fibrosing interstitial lung disease (20). These findings were consistent with ours showing that HE4 was significantly associated with pulmonary involvements and renal involvements in patients with pSS. While it remains unclear whether HE4 is implicated in this process, LeBleu et al. have shown that HE4 can suppress the activity of multiple proteases, including serine proteases and matrix metalloproteinases, and specifically inhibits their capacity to degrade type I collagen, thereby promoting the development of kidney fibrosis (8). Thus, HE4 is likely to exert a pathogenic role in pulmonary and renal damage in pSS. Further studies are needed to define the functional relevance of HE4 in the pathogenesis of pSS.

Another major finding is that we identified a significant correlation between the levels of HE4 with disease activity. pSS patients in our study presented with moderate to high disease activity (defined by ESSDAI ≥5) showed significantly elevated levels of HE4. Our results were consistent with the findings by Ren et al., who showed that serum levels of HE4 were significantly associated with the SLE disease activity index (SLEDAI) (21). Thus, HE4 may be a potential biomarker to monitor disease activity in pSS.

Our study has a number of notable strengths. To the best of our knowledge, our study represents the first study investigating the clinical performance of HE4 in patients with pSS. Our findings thus expand our understanding of the clinical utility of HE4 in clinical practice, especially in rheumatoid diseases, such as pSS. It should be noted, however, that our study has several limitations. First, it was not a prospective study but rather reflecting a real-world experience with routine clinical tests on HE4. Second, it was a single-center study and the sample size was small, thereby predisposing to selection bias. Third, this study lacked histopathology data. Further multi-center prospective study with a larger cohort will be invaluable to enlighten the clinical utility of HE4 in clinical stratification of pSS and other rheumatoid diseases.

In summary, our findings showed that HE4 was markedly elevated in patients with pSS, and positively associated with ESSDAI, pulmonary and renal involvements, suggesting a diagnostic potential of HE4 in clinical stratification in pSS. Our findings also suggesting that introducing HE4 to the current pSS test panel may provide additional diagnostic value to the current clinically available assays.

## Data Availability Statement

The raw data supporting the conclusions of this article will be made available by the authors, without undue reservation.

## Ethics Statement 

The study protocol was reviewed and approved by the Ethical Committee of PKUPH (Protocol number: 2019PHB244). The patients/participants provided their written informed consent to participate in this study.

## Author Contributions 

JC, FS, HB, LL, and MZ: experiments, data acquisition, data analysis. HY, JH, and YL: study design, data analysis, and manuscript preparation. All authors contributed to the article and approved the submitted version.

## Funding

This work was supported in part by grants from National Natural Science Foundation of China, grant no. 81971521, 81801618.

## Conflict of Interest

The authors declare that the research was conducted in the absence of any commercial or financial relationships that could be construed as a potential conflict of interest.
